# Salivary gland carcinoma (SGC) with perineural spread and/or positive resection margin – high locoregional control rates after photon (chemo) radiotherapy - experience from a monocentric analysis

**DOI:** 10.1186/s13014-019-1260-x

**Published:** 2019-04-23

**Authors:** Marlen Haderlein, Claudia Scherl, Sabine Semrau, Sebastian Lettmaier, Markus Hecht, Florian Putz, Heinrich Iro, Abbas Agaimy, Rainer Fietkau

**Affiliations:** 1 Department of Radiation Oncology, University Hospital, Friedrich-Alexander-Universität Erlangen-Nürnberg (FAU), D-91054 Erlangen, Germany; 2 Department of Otorhinolaryngology, University Hospital, Friedrich-Alexander-Universität Erlangen-Nürnberg (FAU), Erlangen, Germany; 3 Institute of Pathology, University Hospital of Erlangen, Friedrich-Alexander-Universität Erlangen-Nürnberg (FAU), Erlangen, Germany; 4 Department of Otorhinolaryngology, University Hospital of Mannheim, Mannheim, Germany

**Keywords:** Salivary gland cancer, Locoregional control, Perineural spread, Positive resection margin, Photon radiotherapy

## Abstract

**Background:**

The aim was to evaluate the outcome, especially locoregional control of patients with locally advanced salivary gland carcinoma (SGC) with perineural spread (Pn1) and/or positive resection margins (R1/2) after postoperative photon (chemo) radiotherapy in a single centre.

**Methods:**

We retrospectively reviewed data of 65 patients with newly diagnosed locally advanced SGC without distant metastases who underwent radio (chemo) therapy in the department of radiation oncology of the university hospital of Erlangen from January 2000 until April 2017.

Kaplan Meier method was used to calculate survival and recurrence rates. In univariate analysis the log-rank test was used to correlate patient−/tumor- and treatment-related parameters to survival and recurrence rates.

**Results:**

Median follow-up was 45 months (range: 6; 215).

After 1, 3, 5 years cumulative incidence of local and locoregional failure was 3.1, 7.0, 7.0% and 3.1, 9.7, 12.9%, whereas cumulative incidence of distant metastases (DM) was 15.6, 36.0, 44.0%. After 1,3, 5 years cumulative Overall (OS) and Disease-free survival (DFS) was 90.5, 74.9, 63.9% and 83.0, 54.8, 49.4%.

The only significant predictor for decreased local and locoregional control was a macroscopic resection margin(R2) (*p* = 0.002 and *p* = 0.04). High-grade histology (*p* = 0.006), lymph node metastases with extracapsular spread (*p* = 0.044) and an advanced T-stage (*p* = 0.031) were associated with an increased rate of DM. High-grade histology was the only factor predicting for a decreased DFS (*p* = 0.014).

**Conclusion:**

Photon radiotherapy leads to high local and locoregional control rates in a high-risk patient population with SGC with microscopically positive resection margins and/or perineural spread. The most common site of disease recurrence was distant metastases. Therefore the real challenge for the future should be to prevent distant metastases.

## Introduction

Salivary gland carcinoma is a rare tumor entity including a variety of different histologic subgroups [1]. Large retrospective studies provide evidence suggesting a benefit of postoperative external beam radiotherapy in locally advanced salivary gland cancer [2, 3]. Especially in patients with perineural invasion and/or positive resection margins locoregional tumor control after surgery is low [2–4]. Moreover adenoidcystic carcinoma (ACC) is known as a radioresistant tumor and in case of macroscopic tumor high LET (linear energy transfer) radiation with protons or especially carbon ions might be beneficial [5–7]. In recent times high LET radiation was also investigated in patients with all kinds of salivary gland carcinomas in case of high-risk features (positive resection margin and/or perineural invasion) in the postoperative situation [8–10].

Of the large retrospective studies that support effectiveness of postoperative photon radiotherapy [11] in patients with locally advanced SGC there is no study that only investigates patients with the high-risk features perineural spread and/or positive resection margins like those patients who met inclusion citeria for participating in trials using carbon ion radiotherapy in the postoperative situation. The purpose of this retrospective monocentric analysis was to evaluate local/locoregional control (LC/LRC) in patients with salivary gland carcinoma with microscopic or grossly positive (R1/R2) resection margins and/or perineural invasion after postoperative radio (chemo) therapy with photons. Therefore we reviewed all patients with salivary gland carcinoma with positive resection margin and/or perineural spread, who underwent postoperative radio (chemo) therapy in our department between 2000 and 2017.

## Material and methods

### Data collection, patient characteristics

We reviewed clinical records of patients with newly diagnosed locally advanced salivary gland carcinoma of the head and neck region who were treated with surgery followed by radio (chemo) therapy from January 2000 until April 2017. Only patients with perineural invasion (Pn1) and/or positive resection margins (R1/R2 resection) were included in the analysis. Moreover patients with distant metastases at the date of first diagnosis, patients with squamous cell carcinoma or patients who underwent brachytherapy alone were excluded.

For detailed patient information see Table 1:

**Table 1:** Patient characteristics

Patients’ characteristics (*n* = 65)		
Characteristics	Value	%
Gender (Number of patients)		
female	30	46.2%
male	35	53.8%
Age at diagnosis (years)		
Median	57	
Range	26–83	
Primary tumor site (Number of patients)		
Parotid Gland	36	55.4%
Submandibular Gland	11	16.9%
Minor Salivary Glands:	18	27.7%
- Nasal cavity/paranasal sinus	6	
- Hard palate	3	
- Tongue/base of tongue	5	
- Meatus acusticus	2	
- Floor of mouth	1	
- Cheek	1	
Histologic Subtypes (Number of patients)		
Adenoidcystic Carcinoma	25	38.5%
Mucoepidermoid Carcinoma	13	20.0%
Ductal Adenocarcinoma	19	29.2%
Adenocarcinoma NOS	4	6.2%
Acinic cell carcinoma	2	3.1%
other	2	3.1%
Tumor stage (Number of Patients)		
pT1	9	13.8%
pT2	10	15.4%
pT3	30	46.2%
pT4	16	24.6%
pT4a	13	
pT4b	3	
Primary tumor size (cm)		
Median:	2.8	
Range:	1–8.5	
Nodal Stage (Number of Patients)		
c/pN0	27	41.5%
pN1	14	21.5%
pN2b	24	36.9%
Perinodal Spread (Number of Patients)		
no	39	60.0%
yes	21	32.3%
unknown	5	7.7%
Lymphangiosis (Number of patients)		
L0	43	66.2%
L1	20	30.8%
Lx	2	3.1%
Hemangiosis (Number of patients)		
V0	52	80.0%
V1	11	16.9%
Vx	2	3.1%
Perineural Spread (Number of Patients)		
Pn0	6	9.2%
Pn1	59	90.8%
Resection margin (Number of Patients)		
R0	40	61.5%
R1	19	29.2%
R2	6	9.2%
Grade of Differentiation (Number of Patients)		
Low-grade	6	9.2%
Intermediate-grade	11	16.9%
High-grade	47	72.3%
Gx	1	1.5%
Bone Infiltration of primary tumor		
No	59	90.8%
Yes	6	9.2%
Neck Dissection (Number of Patients)		
None	10	15.4%
Ipsilateral	48	73.8%
Bilateral	7	10.8%
Radio (chemo) therapy (Number of patients)		
Radiation alone	24	36.9%
Radiochemotherapy	41	63.1%
Radiation dose (Gy):		
Median: 64		
Range: 45–74.2Gy		
Radiation technique (Number of patients)		
3D:	38	58.5%
IMRT:	27	41.5%
Planning target volume (number of patients)		
Primary tumor region	11	16.9%
Primary tumor region, ipsilateral neck	35	53.8%
Primary tumor region, bilateral neck	19	29.2%

### Treatment

All patients were treated with surgery followed by radiotherapy or radiochemotherapy with curative intention. Local resection of primary tumor was performed in all patients. A second primary tumor resection was necessary in 14 patients (21.5%). Resection margin was negative in 40 patients (61.5%) and microscopically positive in 19 patients and 6 patients had a macroscopically positive resection margin in the final pathologic results. 48 (73.8%) patients underwent ipsilateral neck dissection alone, 7 patients (10.8%) underwent bilateral neck dissection and in 10 (15.4%) patients no neck dissection was performed. Forty-one patients (63.1%) received chemotherapy simultaneously. Of these 41 patients 38 patients received platinum-based and 3 patients mitomycin c based chemotherapy. Usually chemotherapy was recommended for patients with locally advanced T-stage T3/4, high grade disease, lymph node metastases with perinodal spread, ≥3 lymph node metastasis or positive/close resection margins. Patients with these tumor characteristics, who did not receive additional chemotherapy, either refused chemotherapy or suffered from comorbidities (1 patient), that did not allow chemotherapy .

The median radiotherapy dose administered was 64 Gy (range: 45Gy; 74.2Gy).

Target volume delineation was performed on a contrast-enhanced computed tomography scan. The preoperative CT and/or MRI scan was fused to the planning CT.

CTV always included the primary tumor region along the nerve tracts up to the base of skull. In case of positive ipsilateral neck nodes ipsilateral lymph nodes were included and if the primary tumor was located at or crossing the midline or patients had multiple ipsilateral lymph node metastases with extracapsular extension, bilateral nodes were included.

After treatment patients had regular follow-up visits in our department as well as in the department of otorhinolaryngology. Usually these visits occurred every 3 months in the first 2 years after treatment, then every 6 months and after 5 years patients had yearly follow-up visits. Usually patients underwent an ENT examination and an ultrasound of the neck at every visit. Every 6 months in the first 5 years patients received an x-ray or CT of the chest (CT usually including the upper stomach). In case of symptoms diagnostic imaging was performed immediately.

### Statistics/analysis of data

Overall survival and disease-free-survival as well as the cumulative incidence of locoregional-recurrence and distant metastases were scored from the time of first diagnosis (defined as the date of biopsy or surgery of the primary tumor). Disease-free survival was defined as the absence of locoregional or distant disease recurrence and death from any cause. Statistical analysis was performed using the SPSS software version 21. The Kaplan-Meier-Method was used for calculating estimated overall-survival, disease-free-survival and the cumulative incidence of local/locoregional control and distant metastases. In univariate analysis the log-rank-test was used to correlate patient- tumor- and treatment-related parameters with overall-survival, disease-free-survival, incidence of local/loregional recurrences and distant metastases. Therefore the following dichotome risk-classification of the different variables was used considering the limited number of patients: gender; age (< 70 years, ≥70 years); adenoidcystic vs. non-adenoidcystic carcinoma; tumor site (parotid vs non-parotid); T-stage(T1/2 vs T3/4); tumor size(≤3 cm vs > 3 cm); N-stage (N0 vs N1/2); number of metastatic lymph nodes: > 1 vs. ≤ 1; extranodal extension; perineural spread; lymphovascular/vascular invasion; high vs. low/intermediate grade; resection margin(R0 vs R1/2; R0/1 vs. R2); neck dissection; number of dissected lymph nodes (≥10 vs. < 10;); Lymph node density (≤4 vs > 4), second primary tumor resection; applied radiation dose (<64Gy vs ≥64Gy), radiation technique (3D vs. IMRT). Only variables yielding *p*-values of ≤0,05 were subsequently included in the multivariate analysis. For multivariate analysis the cox regression analysis was used.

A two-sided p-value <0,05 was considered to be statistically significant.

## Results

Median follow-up was 45 months (range: 6; 215). Of the 65 patients perineural spread was present in 59 patients and positive resection margins in 25 patients, 19 patients showed perineural spread and a positive resection margin.

Of the 65 patients included in the analysis, seven (10.8%) developed local relapse and 28 (43.1%) developed distant metastases .

At the last follow-up, 29 patients (44.6%) were still alive and free from recurrence.

### Local/Locoregional recurrence

**Fig. 1:**
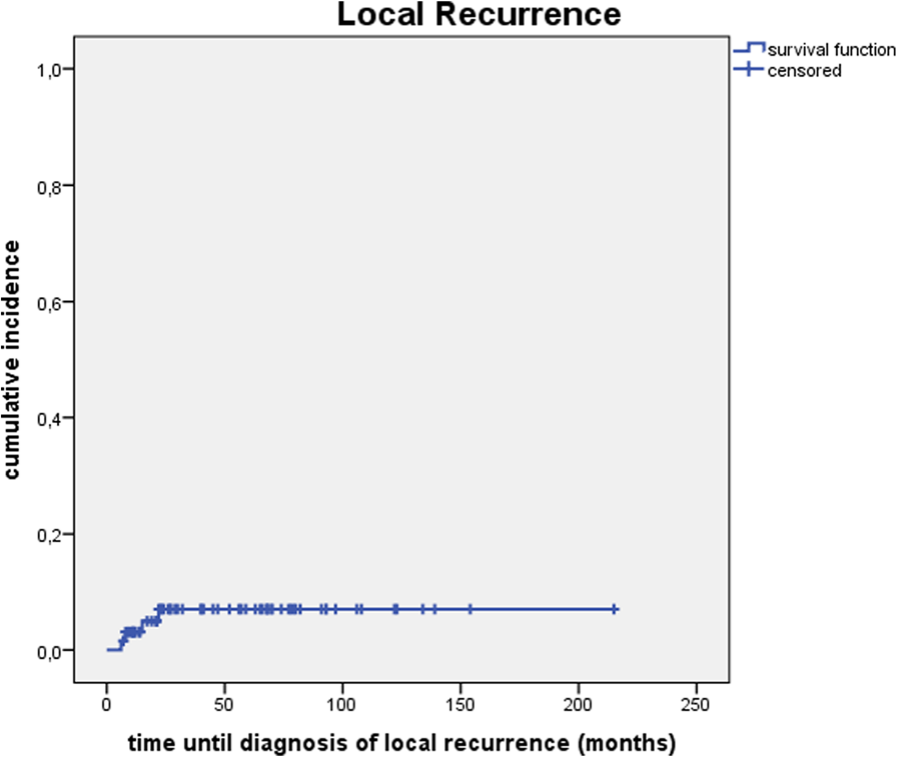
Cumulative incidence of local recurrence

**Fig. 2:**
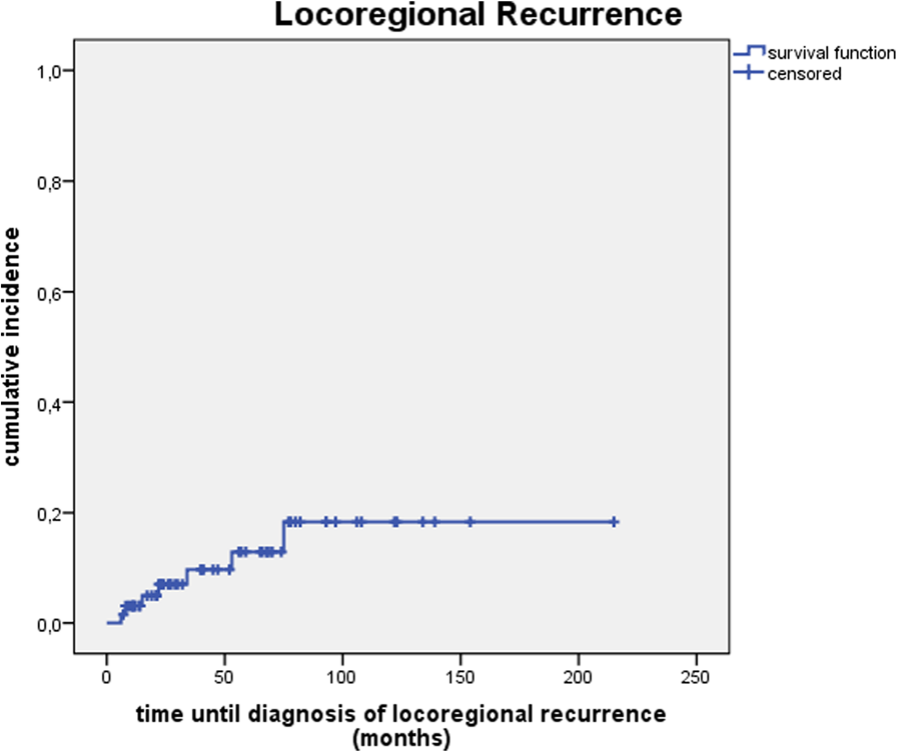
Cumulative incidence of locoregional recurrence

Cumulative incidence of local recurrence (see Fig. 1) and locoregional recurrence (see Fig. 2) was 3.1, 7.0, 7.0% and 3.1, 9.7, 12.9% after 1, 3 and 5 years.

**Fig. 3:**
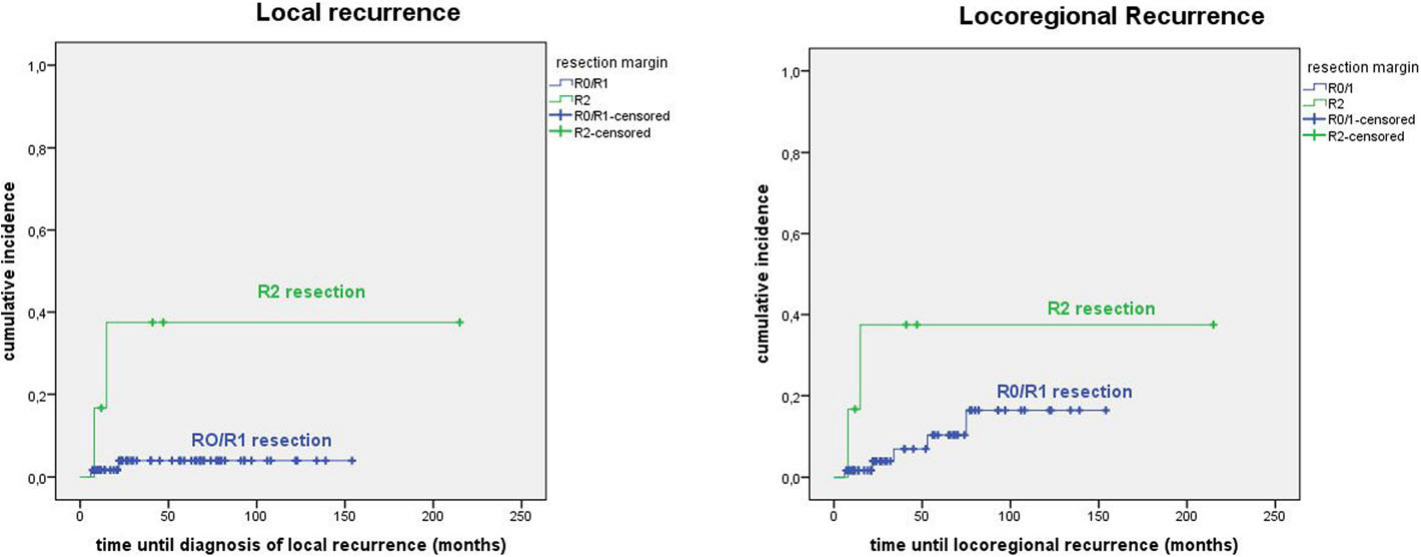
Cumulative incidence of local (3**a**) and locoregional (3**b**) recurrence according to resection margin

The only parameter correlating significantly with the incidence of local (see Fig. 3a) recurrence was a macroscopic resection margin (*p* = 0.002). In univariate analysis gender (*p* = 0.05) and a macroscopic resection margin (*p* = 0.031) were significantly associated with decreased locoregional control. For detailed information see in the Appendix in Table 3. In multivariate analysis only a macroscopic resection margin remained a significant predictor (*p* = 0.04) (see Fig. 3b). Three of the 40 patients with R0 resection, 2 of the 19 patients with microscopic positive resection margin and 2 of the 6 patients with grossly positive resection margin developed a locoregional recurrence. In this context it should be noted that one of the 2 grossly positive resected patients who developed a local recurrence had the R2 situation not in the primary tumor region but in the cervical lymph nodes.

There was a trend towards superior local control for intensity-modulated radiotherapy in comparison to 3D-conformal radiotherapy (*p* = 0.099).

Overall locoregional recurrences were diagnosed in 7 patients. Five of these patients also developed distant metastases, whereas in 2 patients synchronous distant metastases occurred, in one patient distant metastases were already known 18 months before occurrence of locoregional recurrence and in two patients distant metastases were diagnosed after diagnosis of locoregional recurrence. Four of these 7 recurrences were in-radiation-field recurrences while the remaining 2 were outside the radiation field (one in the contralateral lymph nodes, one in the thyroid gland) and one in the margin of the radiation field: the sinus cavernosus (for detailed information see Table 2).

**Table 2:** Locoregional recurrences

Patient	Tumor histology /stage	Localisation of locoregional recurrence	In-Field
Pat.1 (male)	High-grade mucoepidermoid carcinoma of the parotid gland pT3pN1(1/18)L1V1Pn1R0	Gingiva, Mandibula with infiltration of the skin, also disseminated lung metastases at the timepoint of diagnosis of locoregional recurrence	yes
Pat.2 (male)	Ductal adenocarcinoma of the parotid gland, high-grade pT4pN2b(19/38)L1V0Pn1 R2 (detection of supraclavicular lymph node metastases before start of radiochemotherapy)	Recurrence in the parotid region with orbital infiltration, also disseminated bone metastases at the timepoint of diagnosis of locoregional recurrence	yes
Pat.3 (male)	Intermediate-grade mucoepidermoid carcinoma of the parotid gland pT1pN2b(2/3)L0V0Pn0R1	Recurrence in the parotid region (R0 resection of recurrence was possible)	yes
Pat.4 (male)	Low-grade adenoidcystic carcinoma of the submandibular gland pT3pN0(0/34)L0V0Pn1R1	Recurrence in the thyroid gland, diagnosis of lung metastases 13 months after diagnosis of locoregional recurrence	No
Pat.5 (male)	Ductal carcinoma of the parotid gland, high-grade pT4pN2b(22/28)LxVxPn1R0	Contralateral lymph node metastases, diagnosis of lung and liver metastases 6 months after diagnosis of locoregional recurrence	no
Pat.6 (female)	High-grade adenoidcystic carcinoma of the sinus maxillaris on the left side with skull base and orbital infiltration pT4cN1R2	Recurrence in sinus maxillaris	yes
Pat.7 (male)	High grade adenoidcystic arcinoma of the submandibular gland on the left side pT3 pN0 (0/24) cM0 L0 V0 Pn1 R0	Recurrence in Sinus cavernosus, also disseminated lung metastases diagnosed 18 months before diagnosis of locoregional recurrence	Margin of radiation field

### Overall and disease-specific survival

OS was 90.5, 74.9 and 63.9% after 1, 3 and 5 years. Disease-specific survival was 93.6, 80.8 and 71.0% after 1, 3 and 5 years.

### Disease-free survival

**Fig. 4:**
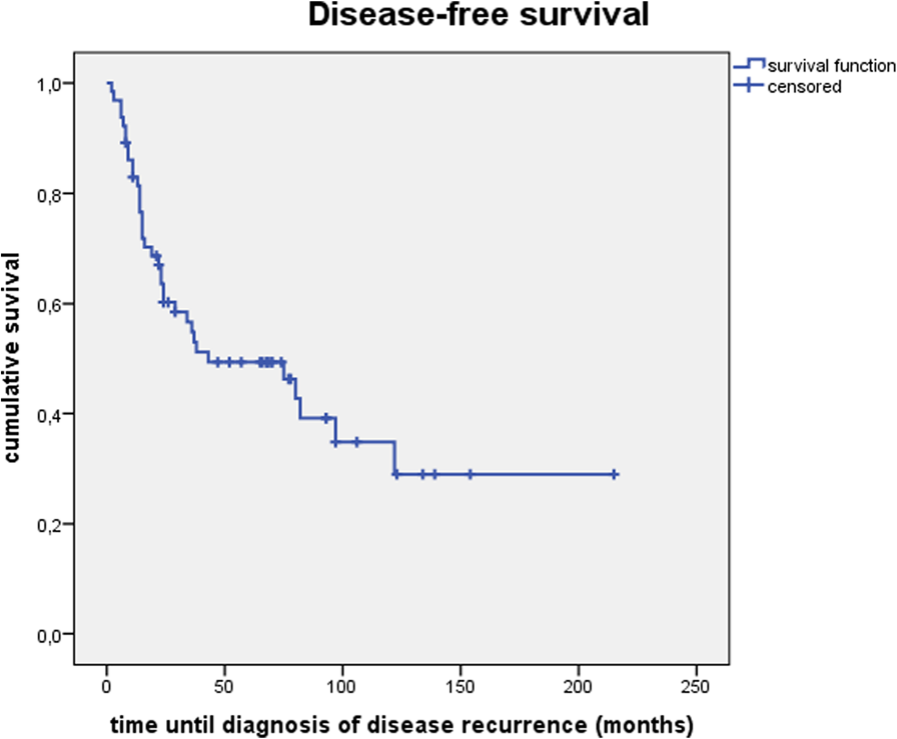
Disease-free survival

DFS was 83.0, 54.8 and 49.4% after 1, 3 and 5 years (see Fig. 4).

In univariate analysis high-grade histology (*p* = 0.014) was the only significant predictor for a decreased DFS. In patients with low-or intermediate grade salivary gland carcinoma DFS was 100, 82.4, 82.4% after 1,3 and 5 years while in patients with high-grade histology DFS was 76.4, 44.0, 35.8% after 1,3 and 5 years.

### Distant metastases

**Fig. 5:**
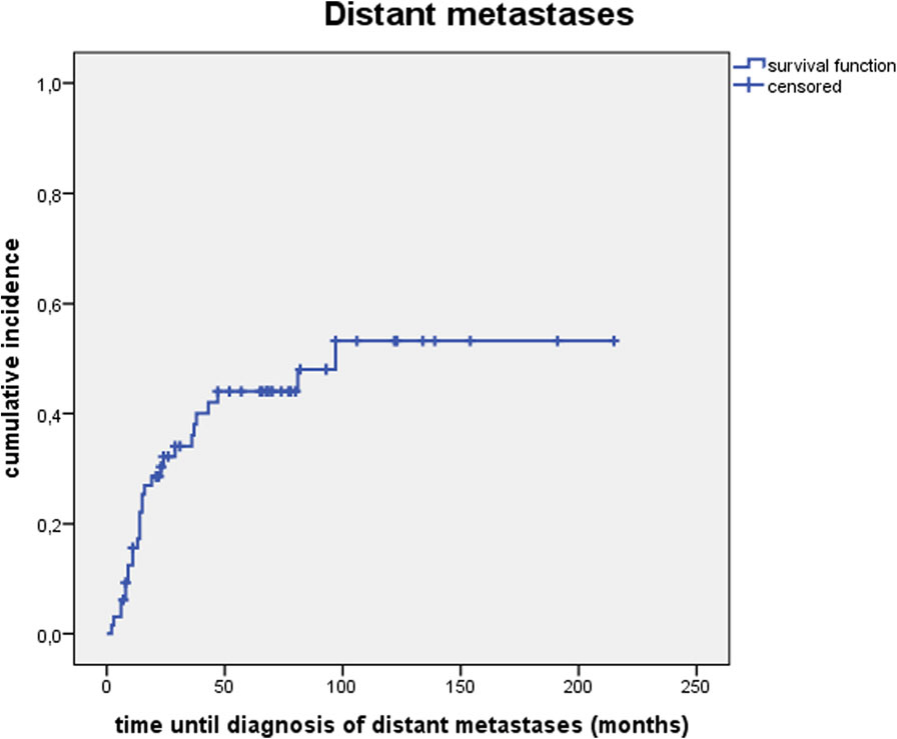
Cumulative incidence of distant metastases

Cumulative incidence of distant metastases was 15.6, 36.0 and 44.0% after 1, 3 and 5 years (see Fig. 5).

Distant metastases were found in the following locations: lung: *n* = 19 patients, bone: *n* = 3 patients, liver: *n* = 2 patients, disseminated: *n* = 4 patients.

In univariate analysis an advanced T-Stage (*p* = 0.031), high-grade histology (*p* = 0.006) and the presence of extracapsular extension of lymph node metastases (*p* = 0.044) was associated with an increased incidence of distant metastases. In multivariate analysis none of the variables reached statistical significance.

## Discussion

This study evaluates the outcome of patients with salivary gland carcinoma (SGC) with positive resection margin and/or perineural invasion after photon (chemo-)radiotherapy. Because of the rarity of SGC no prospective study on the impact of radiotherapy in the postoperative situation is available. There is evidence from large retrospective studies and experience from single centre evaluations that show a benefit of postoperative radiotherapy in patients with SGC with high-risk features [2, 3]. In retrospective studies a positive resection margin and/ or perineural spread were identified as risk factors for locoregional recurrence of salivary gland cancer [2–4].

To the best of our knowledge this is the only retrospective analysis of the impact of photon (chemo) radiotherapy in patients with SGC after surgery, which only includes patients with positive resection margin and/or perineural spread.

This retrospective study shows high local and locoregional control rates of over 93 and 87.1% after 5 years in a high-risk population of patients with salivary gland carcinoma. In 7 out of 65 patients locoregional recurrence occurred.

The COSMIC trial [8–10] investigates the effect of carbon ion radiotherapy in all kinds of SGC including patients with microscopic resection margins defined as microscopically positive resection margin and/or showing perineural spread (referred to as R1-group). Preliminary results of the cosmic trial show a locoregional control rate of 89.7%/ 86.9% and a PFS of 64.6%/58.8% after 3 years in the R1/R2 group compared to a locoregional control rate of 87.1% and a DFS of 54.8% after 3 years in our retrospective analysis.

It has to be mentioned that some patient characteristics were different in the COSMIC trial in comparison to our study. First of all 14 of the 20 patients (70%) in the group with microscopic positive resection margin and all patients of the group with macroscopic positive resection margin of the cosmic trial were patients with adenoidcystic carcinomas. The authors of the study state that “there were no significant differences between ACC and non-ACC histologic features regarding LC, PFS or OS.” In our analysis 38.5% of patients had an adenoidcystic carcinoma, followed by patients with salivary duct carcinoma (29%) and mucoepidermoid carcinoma (20%). The high percentage of salivary duct carcinomas, which are known for their high rate of developing distant metastases [12–14] during follow-up, might explain the decreased disease-free survival in our study. Moreover in our patient population most patients had salivary gland cancer of the parotid gland (55%) whereas in the cosmic trial most of the patients had their primary tumor located in a minor salivary gland (especially paranasal sinus and palate).

Nevertheless, our results show a high local and locoregional control rate in patients with salivary gland carcinoma independent of tumor type and tumor location. The only significant predictor for decreased local and locoregional control was a macroscopic resection margin. There were, however, only 6 patients with an R2 resection in our analysis. Two of these patients developed local recurrence, with one of these patients having the R2 situation not in the primary tumor region, where the recurrence occurred, but in the cervical lymph nodes. Moreover it is worth noting, that the locoregional control rate of our analysis is comparable to that of the R1 group of the COSMIC trial. In the R1 group of the COSMIC trial no patients with macroscopic tumor margin were included.

Despite the high locoregional control rate DFS was only 49.4% after 5 years, because of a high rate of distant failure (44% after 5 years). Interestingly, in the Cosmic trial the first site of failure was also distant (> 50% of the patients). Moreover other retrospective trials [15–17] report high rates of distant metastases in up to 47.3% of patients with salivary gland cancer, especially in high-grade carcinomas. Considering the high rates of locoregional control it is doubtful whether carbon ion radiotherapy is superior to photon radiotherapy in patients with salivary gland cancer with microscopically positive resection margins and/or perineural spread. In patients with macroscopic resection margin carbon ion radiotherapy might be considered.

Intensity-modulated radiotherapy showed a trend towards improved local control when compared to 3D-conformal radiation. None of the patients in the IMRT group developed a local recurrence, but follow-up is shorter in this group than in the 3d-conformal group.

The implementation of IMRT in recent years in combination with image-guided radiotherapy and their continuous development has led to the possibility of dose escalation in the tumor region while simultaneously reducing radiation dose at the organs at risk [18–25]. Especially in critical regions like the skull base highly conformal radiotherapy with IMRT might be beneficial [26, 27]. This means that in our patient population local control might even have been higher, if all patients had been treated with IMRT. Patients that were treated with 3D-conformal radiotherapy are not comparable to patients treated with carbon ion radiotherapy.

In this analyses high-grade histology is the only significant predictor for a decreased DFS as also reported in a previous study [28]. Furthermore patients with high-grade histology, an advanced T-stage and the presence of extracapsular extension of lymph node metastases show a significant higher rate of distant metastases during follow-up. These findings are in line with the literature [4, 16, 28]. An advanced T-Stage is associated with a higher rate of distant metastases but not with a decreased local control. It may therefore be assumed that in patients with an advanced T-stage und additional risk factors like positive resection margin and/or perineural spread, the risk of development of distant metastases is much higher than that of experiencing locoregional recurrence.

Laramore et al. [29] in their final report on the RTOG trial comparing definitive neutron and photon radiotherapy stated, that local control was increased by neutron therapy, but that this did not translate into an improved overall survival, because of the high rate of distant metastases during follow-up.

In future the real challenge will therefore be to investigate intensified first-line systemic treatments to decrease the rate of distant metastases.

It also has to be mentioned, that this study has several limitations. First of all the evaluation we performed was retrospective with a small number of patients and with a heterogeneous mix of different tumor subtypes and sites, which makes the evaluation of treatment outcome more difficult. Moreover because of the retrospective nature of our study the times at which radiologic imaging was performed were not clearly defined and might have influenced the survival analysis. Radiation techniques have changed over the last few years. The implementation of intensity-modulated radiotherapy in combination with image-guided radiotherapy has led to higher treatment accuracy and to the possibility of applying higher doses to the tumor region. Therefore the fact that we included patients, who were irradiated using 3D-conformal techniques may have led to lower locoregional control rates in our study. It is doubtful if a multivariate analysis makes sense considering the small number of patient. Toxicity was not assessed. This could be a potential advantage ofproton or carbon ion radiotherapy over radiotherapy with photons.

Despite these shortcomings this retrospective study shows that in patients with salivary gland carcinoma high local and locoregional control rates are achieved even in patients with positive microscopic resection margins and/or perineural spread after postoperative radiotherapy with photons. In the future the real challenge in the treatment of salivary gland cancer will be the optimization of systemic treatment options to decrease distant failure.

## Conclusion

Photon radiotherapy leads to high local and locoregional control rates in a high-risk patient population with SGC with microscopically positive resection margins and/or perineural spread. The most common site of disease recurrence was distant metastases. Therefore the real challenge for the future should be to prevent distant metastases.

## Appendix

**Table 3:** Patient-, tumor-and treatment-related factors compared to local and locoregional recurrences

Univariate		
	***Local recurrence*** ***p-value*** Hazard ratio (95% confidence interval)	***Locoregional recurrence*** ***p-value*** Hazard ratio (95% confidence interval)
Patient-dependent		
Gender	**0.389**	**0.050**
female vs. male	0.38 (0.04–3.69)	0.16 (0.02–1.31)
Age at diagnosis	**0.839**	**0.828**
≥70y vs. <70y	1.27 (0.13–12.24)	0.79 (0.09–6.68)
Tumor-related		
Primary tumor site	**0.368**	**0.664**
non-parotid vs. parotid	0.37 (0.04–3.54)	0.72 (0.16–3.23)
Histologic Subtype	**0.541**	**0.953**
non-adenoidcystic vs.	2.00	0.956
adenoid cystic	(0.21–19.21)	(0.21–4.29)
T classification	**0.773**	**0.196**
T3/4 vs. T1/2	1.39 (0.15–13.43)	3.75 (0.44–31.85)
Primary tumor size, cm	**0.703**	**0.711**
>3 cm vs. ≤ 3 cm	0.63 (0.06–6.95)	1.35 (0.27–6.72)
N classification	**0.075**	**0.227**
N1/2 vs. N0	53.2 (0.02–180,704.08)	2.68 (0.51–14.09)
Number of metastatic lymph nodes	**0.285**	**0.224**
>1 vs. ≤ 1	3.42 (0.31–37.85)	2.68 (0.52–13.88)
Histological tumor grade	**0.747**	**0.537**
High vs. low/intermediate	1.20 (0.39–3.75)	1.30 (0.56–2.99)
Extranodal extension	**0.228**	**0.581**
yes vs. no	0.03 (0.00–656.48)	0.55 (0.06–4.78)
Perineural Spread	**0.222**	**0.403**
yes vs. no	0.27 (0.03–2.60)	0.41 (0.05–3.51)
Lymphovascular invasion	**0.163**	**0.587**
yes vs. no	4.72 (0.43–52.22)	1.64 (0.27–9.82)
Vascular invasion	**0.380**	**0.680**
yes vs. no	2.80 (0.25–31.05)	1.58 (0.18–14.23)
Bone invasion	**0.222**	**0.603**
yes vs. no	3.72 (0.39–36.03)	1.75 (0.21–14.60)
Treatment-related		
Resection margin	**0.119**	**0.179**
R1/2 vs. R0	5.04 (0.52–48.53)	2.81 (0.62–12.72)
Resection margin	**0.002**	**0.031**
R2 vs R0/1	11.26 (1.58–80.45)	5.15 (0.98–26.93)
Second primary tumor resection	**0.293**	**0.543**
yes vs. no	0.034 (0.00–910.95)	0.52 (0.06–4.37)
Neck Dissection	**0.598**	**0.852**
yes vs. no	0.55 (0.06–5.28)	1.22 (0.15–10.18)
Number of dissected nodes	**0.675**	**0.708**
≥10 vs. < 10	0.60 (0.05–6.64)	1.50 (0.18–12.88)
Lymph node density (%)	**0.110**	**0.246**
>4 vs. ≤4	56.27 (0.01–637,456.03)	2.71 (0.48–15.42)
Radiation dose	**0.333**	**0.698**
≥64Gy vs. <64Gy	0.34 (0.04–3.31)	0.66 (0.08–5.50)
Planning target Volume	**0.318**	**0.108**
primary tumor region &	28.57 (0.00–1,127,372.02)	34.53 (0.03–47,010.85)
regional lymph drainage vs.		
primary tumor region		
Radiation technique	**0.099**	**0.226**
IMRT vs. 3D	0.02 (0.00–82.02)	0.290 (0.03–2.45)

## Abbreviations

ACC: Adenoidcystic carcinoma
CT: Computer tomography
DFS: Disease-free survival
DM: Distant metastases
IMRT: Intensity-modulated radiotherapy
LC: Local control
LET: Linear energy transfer
LRC: Locoregional control
OS: Overall survival
PFS: Progression-free survival
Pn1: Perineural spread
R0 resection: complete resection
R1 resection: microscopically positive resection margins
R2 resection: macroscopically positive resection margins
SGC: Salivary gland cancer
